# Role of *FaSOC1* and *FaCO* in the seasonal control of reproductive and vegetative development in the perennial crop *Fragaria* × *ananassa*

**DOI:** 10.3389/fpls.2022.971846

**Published:** 2022-08-17

**Authors:** Julio C. Muñoz-Avila, Concepción Prieto, José F. Sánchez-Sevilla, Iraida Amaya, Cristina Castillejo

**Affiliations:** ^1^Laboratorio de Mejora y Biotecnología, Instituto Andaluz de Investigación y Formación Agraria y Pesquera (IFAPA) Centro de Málaga, Málaga, Spain; ^2^Unidad Asociada de I + D + i IFAPA-CSIC, Biotecnología y Mejora en Fresa, Málaga, Spain

**Keywords:** flowering, runnering, photoperiod, *Fragaria*, cultivated strawberry, *FaSOC1*, gibberellins, *FaCO*

## Abstract

The diploid woodland strawberry (*F. vesca*) represents an important model for the genus *Fragaria*. Significant advances in the understanding of the molecular mechanisms regulating seasonal alternance of flower induction and vegetative reproduction has been made in this species. However, this research area has received little attention on the cultivated octoploid strawberry (*F.* × *ananassa*) despite its enormous agronomical and economic importance. To advance in the characterization of this intricated molecular network, expression analysis of key flowering time genes was performed both in short and long days and in cultivars with seasonal and perpetual flowering. Analysis of overexpression of *FaCO* and *FaSOC1* in the seasonal flowering ‘Camarosa’ allowed functional validation of a number of responses already observed in *F. vesca* while uncovered differences related to the regulation of FaFTs expression and gibberellins (GAs) biosynthesis. While FvCO has been shown to promote flowering and inhibit runner development in the perpetual flowering H4 accession of *F. vesca*, our study showed that *FaCO* responds to LD photoperiods as in *F. vesca* but delayed flowering to some extent, possibly by induction of the strong *FaTFL1* repressor in crowns. A contrasting effect on runnering was observed in FaCO transgenic plants, some lines showing reduced runner number whereas in others runnering was slightly accelerated. We demonstrate that the role of the MADS-box transcription factor FaSOC1 as a strong repressor of flowering and promoter of vegetative growth is conserved in woodland and cultivated strawberry. Our study further indicates an important role of FaSOC1 in the photoperiodic repression of FLOWERING LOCUS T (FT) genes *FaFT2* and *FaFT3* while *FaTFL1* upregulation was less prominent than that observed in *F. vesca*. In our experimental conditions, FaSOC1 promotion of vegetative growth do not require induction of GA biosynthesis, despite GA biosynthesis genes showed a marked photoperiodic upregulation in response to long days, supporting GA requirement for the promotion of vegetative growth. Our results also provided insights into additional factors, such as FaTEM, associated with the vegetative developmental phase that deserve further characterization in the future.

## Introduction

Strawberry (*Fragaria* spp.) belongs to the Rosaceae family and comprise different species among which, the commonly called woodland strawberry *F. vesca* (2n = 2× = 14) has become a genetic model for studying perennial development in Rosaceae (Hytönen and Kurokura, 2020). The cultivated strawberry, *Fragaria* × *ananassa* (2n = 8× = 56), is the most important berry crop in the world, with a global production over 8.8 M tones in 2020 (FAOSTAT 2022). Both species are closely related as an ancestor of *F. vesca* is the dominant progenitor of the octoploid strawberry (Edger et al., 2019). Breeding efforts in *F.* × *ananassa* are challenged by its octoploid nature and a general high heterozygosity, which result in many alleles contributing to trait variation and in the necessity of vegetative propagation to ensure the maintenance of superior genotypes.

Strawberries are perennial rosette plants that can be propagated both sexual (flowering) and asexually (vegetatively or clonally). Vegetative reproduction takes place through runners, also called stolons, which are elongated stems that grow horizontally above ground from which daughter plants arise. They are important for growers and breeders as they are used to propagate strawberry cultivars avoiding trait loss through recombination. However, runnering is also associated with a decrease in fruit yield, as it reduces the number of fruit-bearing shoots (Gaston et al., 2013; Heide et al., 2013; Tenreira et al., 2017). As a consequence, commercial strawberry fruit production is highly influenced by the balance between the two reproductive modes. One of the most important goals in strawberry breeding programs is increasing or maintaining high yield. A better understanding of the genetic and molecular factors that modulate the switch between inflorescence and runner development would facilitate the development of new cultivars with extended fruiting period and/or better adapted to local environments and changing climatic conditions, enabling fruit production under different photoperiods, temperatures or latitudes.

The flowering/runnering decision in strawberry depends on the fate of plant meristems. The stem or crown consists of short internodes produced from the shoot apical meristem (SAM). Each node harbors one leaf and an axillary meristem (AXM). Inflorescences are always formed terminally from the apical meristem (Darrow, 1966; Guttridge, 1985). After the emergence of the terminal inflorescence the crown vegetative extension continues from the uppermost lateral meristem, from which secondary and tertiary inflorescences could arise. On the other hand, a bud emerged from an AXM might remain dormant or activated to grow and develop either into a runner or into a new branch crown, which eventually can bear a terminal inflorescence (Heide et al., 2013; Perrotte et al., 2016b). The fate of the apical and axillary meristems is dictated by genetic and environmental conditions (Heide, 1977; Guttridge, 1985; Sønsteby and Nes, 1998; Bradford et al., 2010; Hytönen and Kurokura, 2020; Andrés et al., 2021).

Cultivated and woodland strawberries are classified based on their photoperiodic response into either seasonal (short-day; SF) or perpetual (day-neutral; PF) flowering types (Iwata et al., 2012; Gaston et al., 2013; Heide et al., 2013; Hytönen and Kurokura, 2020). During the fall, in response to short days (SD) and low temperatures, branch crowns from seasonal types emerge from AXMs and floral induction occurs at the SAMs of the main crown and older branch crowns. After adequate time in flowering-inducing conditions, plants become semi-dormant until sufficient winter chilling resumes vigorous growth. Then, terminal inflorescences emerge from previously induced meristems. Later on, under the long photoperiods (LD) and warm temperatures of summer, strawberry plants grow vegetatively and axillary buds differentiate into runners instead of branch crowns (Guttridge, 1958; Jonkers, 1965; Heide et al., 2013). Additionally, natural PF mutants in which flowering occurs all along the vegetative cycle have been identified in cultivated (*F. × ananassa*) and woodland (*F. vesca*) strawberries (Iwata et al., 2012; Gaston et al., 2013). Heide et al. (2013) studied flowering habit of PF types of woodland and cultivated strawberries in SD and LD conditions and concluded that they could be considered quantitative LD plants rather than day-neutrals.

The genetic control of photoperiodic flowering in both diploid woodland strawberry and octoploid cultivated strawberry have long been investigated (Brown and Wareing, 1965; Gaston et al., 2013). In *F. vesca*, genes affecting natural variation on photoperiodic flowering and runnering have been identified. Two groups independently found recessive mutations in the *F. vesca* homolog of *TERMINAL FLOWER 1* (*FvTFL1*) as the underlying variation in the SF locus (SFL) on chromosome 6, causing perpetual flowering (Iwata et al., 2012; Koskela et al., 2012). The Runnering (R) locus on chromosome 2 causes runnerless plants and a deletion in the active site of the gibberellin (GA) biosynthetic gene *FvGA20ox4* was identified in all *r* mutants (Tenreira et al., 2017). A later study confirmed that FvGA20ox4 is indispensable for runner development and under tight environmental regulation (Andrés et al., 2021). In cultivated strawberry, the PF trait has been shown to be controlled by the major quantitative trait locus (QTL) *FaPFRU*, which has been mapped to chromosome 4B (Gaston et al., 2013; Castro et al., 2015; Perrotte et al., 2016a). The underlying gene for *FaPFRU*, different from *TFL1*, is still unknown and should act as a dominant and positive regulator of flowering (Perrotte et al., 2016a). However, the strong floral repressor role of TFL1 was confirmed in the cultivated strawberry, as silencing of *FaTFL1* in a SF cultivar was sufficient to induce PF (Koskela et al., 2016). Although the *FaPFRU* QTL also displays important negative effects on runnering (Perrotte et al., 2016a), the genetic basis of runnering in cultivated strawberry appears to be complex, as weak additive genetic effects and many small effect QTLs have been detected (Simpson and Sharp, 1988; Hossain et al., 2019).

As in other species, besides photoperiod, temperature has an important effect on flowering initiation in strawberry (Heide, 1977; Sønsteby and Heide, 2006; Heide et al., 2013; Rantanen et al., 2015). In both SF and PF genotypes, flowering can be promoted at temperatures below 10–13°C, behaving essentially as day-neutral, while at temperatures above 23–25°C flowering can be inhibited even under inductive photoperiods. In *F. vesca*, this temperature-dependent induction or repression of flowering relies on FvTFL1, which expression is regulated by ambient temperature, while in mild conditions, *FvTFL1* is under photoperiodic control (Rantanen et al., 2015; Koskela et al., 2017; Hytönen and Kurokura, 2020).

The model plant *Arabidopsis* perceives the increase in day length by a complex mechanism that involves the transcriptional and post-transcriptional regulation of the B-Box transcription factor CONSTANS (CO), which eventually triggers the expression of *FT* at the correct season. FT induction occurs in the leaf companion cells and it is transported through the phloem to the apical meristem, changing the fate of the tissue (Suárez-López et al., 2001; Valverde et al., 2004; Valverde, 2011). This CO–FT module works in most species analyzed, however, the final effect of this signal diverges in different species. For example, in rice, a SD plant, the CO-FT module functions as an activator in SD but a repressor in non-inductive LDs (Hayama et al., 2003). By contrast, in legumes such as *Medicago* or pea, CO-like genes may not have any role in the integration of flowering in response to photoperiodic cues (Serrano-Bueno et al., 2017).

Most of what we know about the molecular events that regulate flowering and runnering in strawberry comes from studies using the diploid model *F. vesca*, in which the LD-activated FvFT1–FvSOC1–FvTFL1 module has a pivotal role in the repression of flowering, in a *FvTFL1* wild type (WT) background, or activation of flowering, in a *fvtfl1* mutant background (Hytönen and Kurokura, 2020). FvCO protein has been suggested as part of the photoperiod measurement system necessary for *FvFT1* induction. In the PF *F. vesca* accession Hawaii-4 (H4), which lacks a functional allele of the floral repressor *FvTFL1*, overexpression of *FvCO* leads to *FvFT1* induction in leaf that correlates with upregulation of SUPPRESSOR OF THE OVEREXPRESSION OF CONSTANS1 (*FvSOC1*) and the floral meristem identity gene APETALA1 (*FvAP1*) at the SAMs, and eventually promotes flowering under LD, whereas silencing of *FvCO* has the opposite effect (Koskela et al., 2017). In SF genotypes, upregulation of *FvTFL1* by FvSOC1 prevents flower induction under LD conditions (Mouhu et al., 2013). In addition, *FvSOC1* promotes vegetative growth and runnering by activating the expression of several gibberellin (GA) biosynthesis genes, including *FvGA20ox4*.

Three *FT* genes have been detected in woodland and cultivated strawberry. In *F. vesca*, induction of *FvFT1* in leaves is regulated by light quality in addition to photoperiod (Koskela et al., 2012; Rantanen et al., 2014; Prisca et al., 2022). Leaf-expressed FvFT2 has been shown to act as a mobile signal for flowering under SD photoperiod, while *FvFT3* was not detected in leaf and may promote plant branching, thus increasing flower number and yield in *F. vesca* (Gaston et al., 2021). Expression analyses of *FaTFL1*, *FaFT1*, *FaFT2*, and *FaFT3* in *F.* × *ananassa* SF cultivars grown under different photoperiods and temperatures suggest that *FaFT1/FaTFL1* and *FaFT3* play important roles on the environmental repression and induction of flowering, respectively (Nakano et al., 2015; Koembuoy et al., 2020).

The tradeoff between vegetative propagation and flowering in both cultivated and woodland strawberries is also regulated by gibberellins (GAs). Exogenous GA application to SD-grown strawberry can mimic the effect of LD conditions, promoting runner initiation and inhibiting flowering (Thompson and Guttridge, 1959; Nishizawa, 1993; Black, 2004). On the other hand, treatment with the GA biosynthesis inhibitor prohexadione-calcium (Pro-Ca) enhances branch crown at the expense of runner formation (Nishizawa, 1993; Black, 2004; Hytönen et al., 2009). These observations were genetically supported in *F. vesca* with the identification of a 9-bp deletion in the GA20-oxidase gene *FvGA20ox4* as the runnerless (r) mutation (Tenreira et al., 2017). The importance of GA signaling for runner production in *F. vesca* was further demonstrated when the DELLA protein FvRGA1, a negative regulator of GA signaling, was identified as a key protein controlling runner formation and the causal loci behind the suppressor of runnerless (srl) mutation (Caruana et al., 2018; Li et al., 2018).

As aforementioned, genetic analysis of the PF trait in *F. vesca* and *F.* × *ananassa* led to the identification of different loci in each species, suggesting that differences in flowering time regulation may exist between them. At the same time, the function of factors like the flowering repressor TFL1 is conserved between the diploid model *F. vesca* and *F.* × *ananassa* (Koskela et al., 2016). In order to establish how much of the knowledge acquired in *F. vesca* can be translated to the commercially cultivated species, the role of other proteins such as FaCO and FaSOC1, as well as additional regulation of flowering, need to be further investigated in the octoploid. It is still unclear whether FT2 or FT3 act as mobile or SAM-based floral promoters under SD conditions in cultivated strawberry. In this work, using a combination of expression analysis of selected flowering time genes and functional validation by transgenesis, we have been able to confirm some genetic responses to photoperiod already described in *F. vesca* but also to uncover differences in the mechanisms regulating the transition to flowering, particularly in aspects related to GA metabolism and signaling.

## Materials and methods

### Plant material and growth conditions

All *F.* × *ananassa* plants used in this study were maintained and grown in shaded greenhouses (standard or for GMOs in the case of transgenic plants) under natural sunlight and temperature conditions at IFAPA, Málaga, Spain. Temperature conditions were recorded in an outdoor meteorological station 1500 m away from the shaded greenhouses. For the different experiments, plants were clonally propagated from runners during June-September and potted in October/November in 22 cm pots with a mixture of universal substrate and river sand (3:1 v/v).

For analysis of gene expression in the different plant tissues, leaf, root, crown, flower and green, white and ripe fruit were sampled as shown in Supplementary Figure 1. They were sampled under SD conditions (9 h38 min/14 h21 min day/night; average maximum and minimum temperature from previous 4 weeks: 17.7/9.7°C) from cultivar Chandler. Three biological replicates were collected, each consisting of 4–6 plants.

For comparison of short day and long day (SD/LD) conditions, leaf and crown samples from seasonal flowering (SF) cultivar Chandler and perpetual flowering (PF) cultivar Selva were harvested in December 21st 2012 (9 h38 min/14 h21 min day/night; average maximum and minimum temperature from previous 4 weeks: 17.7/9.7°C) and June 21st 2013 (14 h40 min/9 h19 min day/night; average maximum and minimum temperature from previous 4 weeks: 26.7/16.5°C) for SD and LD photoperiods, respectively. Three biological replicates were collected at Zeitgeber time (ZT) 6, each consisting of 4–6 plants.

For analysis of circadian rhythmic expression, leaf samples from ‘Chandler’ and ‘Selva’ were collected in two separate experiments, at SD and LD conditions. SD sampling took place on December 22nd 2013 (9 h38 min/14 h21 min day/night; average maximum and minimum temperature from previous 4 weeks: 18.2/8.8°C) and LD on June 23rd 2014 (14 h40 min/9 h19 min day/night; average maximum and minimum temperature from previous 4 weeks: 29.6/19.4°C). The first time point was sampled at dawn and then at 4, 8, 12, 16, 20, and 24 h. Three biological replicates consisting of leaves from at least three plants were collected at each time point.

For evaluation of transgenic phenotypes, 9 clones of each transgenic line were propagated from runners as described above. Nine clones from two pGUS lines (pGUS2 and pGUS3) and from Camarosa wild type (WT) were grown and used as controls. For gene expression studies in 35S:*FaSOC1* lines, young leaf (first leaf with fully expanded leaflets) and crown tissues from 3 biological replicates were collected under natural SD conditions (November 29th 2017, 9 h52 min/14 h17 min day/night; average maximum and minimum temperature from previous 4 weeks: 21.1/10.6°C) at ZT 3, each replicate being a pool from 3 plants. As initial evaluation of T_0_ transgenic plants revealed similar phenotypes in lines pGUS2, pGUS3, and WT non-transformed plants, only the pGUS3 control was kept as control for gene expression studies.

Phenotypic data were analyzed using one-way ANOVA followed by Tukey’s test. A value of *P* < 0.05 was considered as statistically significant. Graphs and statistical analyses were performed using the GraphPad Prism 8 software.

### Gene expression analysis

Total RNA was extracted from three biological replicates following a CTAB protocol (Gambino et al., 2008) from 200 mg of frozen powdered samples from vegetative tissues or 300 mg from fruit samples. Residual DNA from RNA samples was removed using Invitrogen™ TURBO DNA-free™ Kit and cDNA was synthesized from 1 μg of RNA using the High-Capacity cDNA Reverse Transcription Kit (Thermo Fisher Scientific). Gene expression levels were analyzed in triplicate by quantitative real-time PCR (qPCR) with the SsoFast EvaGreen supermix (Bio-Rad) and a CFX96 real-time PCR system (Bio-Rad) using a standard two-step program of 40 cycles, annealing at 60°C. The relative expression was calculated using the geometric mean of the housekeeping genes DBP and GAPDH for normalization and the 2^–ΔΔ*Ct*^ method (Pfaffl, 2001) unless stated otherwise in the figure legend. Primer sequence information for all analyzed genes is listed in Supplementary Table 1.

GraphPad Prism was used for statistical analysis. If data points passed the D’Agostino-Pearson omnibus K2 normality test (*P* < 0.001) an ordinary one-way ANOVA was employed followed by either a Fisher’s LSD test to compare preselected pairs of columns (SD/LD genotypes and GA treatment experiments), or a Tuckey’s test to compare each column mean with every other mean (tissue expression) or, when comparing each group to a control group, a Dunnett’s test (expression in transgenic lines). When data points didn’t pass the normality test, *p*-values were determined by a Kruskal Wallis test, followed by Dunn’s post-test for multiple group comparisons. Mean and SEM of the 3 biological replicates were plotted. *P < 0.05; ^**^P < 0.01; ^***^P < 0.001; ^****^P < 0.0001.

### Plasmid construction

For overexpression of *FaCO* and *FaSOC1*, ORFs were cloned from cv. Selva leaf and crown tissues, respectively. PCR amplification was performed using Pfu polymerase 5-prime and DNA fragments cloned in pGEMT-easy (Promega) and sequenced. To generate the *35S:CO* and *35S:FaSOC1* overexpression constructs, each gene was cloned into the *Sal*I and *Xba*I restriction sites of the pBINPLUS vector (van Engelen et al., 1995). A *35S:GUS* construct in the same pBINPLUS vector was used as control.

### Transgenic plants

Leaf explants from *in vitro* ‘Camarosa’ plants were transformed with *Agrobacterium tumefaciens* LBA4404 harboring the *35S:FaCO, 35S:FaSOC1* or *35S:GUS* constructs as previously described (El Mansouri et al., 1996). Transgenic shoots were selected on medium containing 50 mg/L kanamycin and 500 mg/L carbenicillin and grown *in vitro* in a culture chamber under cool-white light (at 15 mE) and a long-day photoperiod (16-h light/8-h dark) at 22°C. Resistant plantlets were acclimated to soil conditions, then transferred into 22 cm pots and grown in confined greenhouse under natural environmental conditions. Preliminary evaluations on the first year were performed with only one replicate of each transgenic line. For successive years, plants were propagated during the summer and 9 plants of each line and controls were transferred to the greenhouse. Phenotype evaluation was performed during the growing season, from December to September.

### Gibberellin treatment

Gibberellin treatment was performed on 6 weeks old clonally propagated *F.* × *ananassa* ‘Camarosa’ plants grown under natural greenhouse conditions. Six plants were used for each of the 3 biological replicates. GA_3_ (SIGMA S7645) was first dissolved in ethanol at 50 mg/mL and a diluted 1:1000 working solution was made in 0.1% Tween-20 (GA_3_ final concentration 50 mg/L). Controls were treated with a 0.1% Tween-20 0.1% ethanol solution. Plants were sprayed to runoff twice, with the first application conducted at day 0 and a second one at day 2. Crown and young leaf samples were collected at day 3 (October 7th, 11 h41 min/13 h18 min day/night, average maximum and minimum temperature from previous 4 weeks: 26.4/16.4°C) at ZT 3.

## Results

### Photoperiodic regulation of flowering time genes in seasonal flowering and perpetual flowering *F.* × *ananassa* cultivars

**FIGURE 1:**
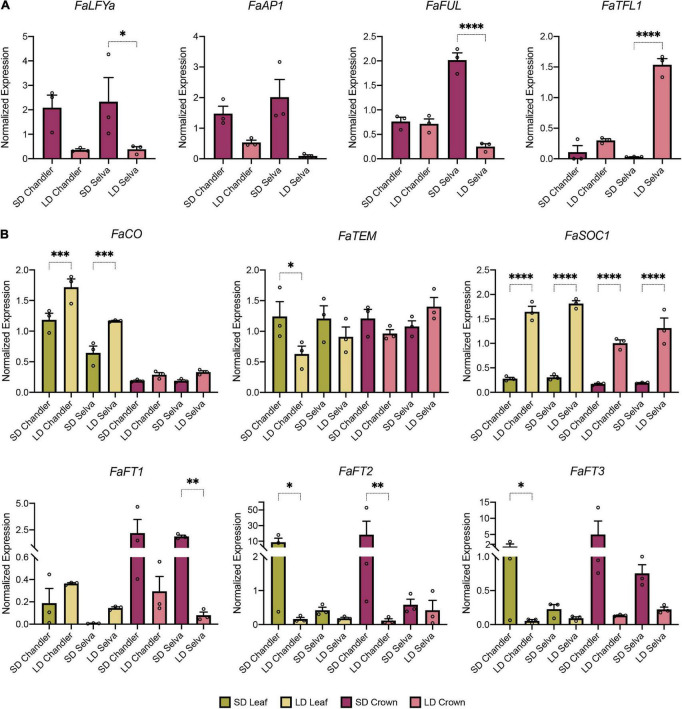
Transcriptional response of flowering time genes to short (SD) and long (LD) days in the seasonal flowering (SF) cultivar Chandler and the perpetual flowering (PF) cultivar Selva. **(A)** Expression of floral meristem identity genes and *FaTFL1* in crowns. **(B)** Expression of flowering time genes in leaves (green) and crowns (purple). Expression was analyzed by qRT-PCR. Graphs show the average of normalized values from three biological replicates ± SEM. In each bar, dots represent the normalized value of each biological replicate. *p < 0.05, **p < 0.01, ***p < 0.001, ****p < 0.0001.

As a starting point for our study on the genetic pathways controlling the transition to flowering in response to day length in cultivated strawberry, we compared the expression of selected flowering time genes in the SF and PF cultivars Chandler and Selva, respectively. Light and photoperiod sensing occurs mainly in leaves and this signal is transmitted to plant meristems to determine their fate (Song et al., 2015). The strawberry plant shoot, also called crown, contains the SAM at terminal position and AXMs at the basis of each leaf. Expression analyses were performed in these two tissues, leaves and crowns (Supplementary Figure 1), collected under natural SD or LD photoperiods. The phenological phase of the meristems was determined by evaluating the expression of the major floral meristem identity genes *FaLFYa, FaAP1*, and *FaFUL*, as well as the repressor of floral transition *FaTFL1* (Figure 1A). Expression levels of *FaLFYa* and *FaAP1* were higher in crowns from plants collected under the inductive SD photoperiods in both cultivars compared to LD, whereas *FaFUL* induction was only detected in crowns from ‘Selva.’ On the other hand, the floral inhibitor *FaTFL1* was downregulated in SD crowns, coinciding with *FaAP1* and *FaLFY* induction. However, *FaTFL1* upregulation under LD was only observed in crowns from ‘Selva.’ Rather than cultivar differences in the signaling pathways, these results are more likely to reveal differences in the timing of phase transitions between them. Altogether, these results suggest that only plants grown in SD presented floral meristems in their crowns, and that the transition to flowering, and later in LD to the vegetative phase, lagged behind in ‘Chandler’ compared to ‘Selva.’ Additionally, the floral meristem identity genes were not upregulated in the LD sampled meristems from the PF cultivar ‘Selva,’ indicating that the transition to the second reproductive phase had not taken place when samples were collected in late June.

In leaf samples harvested under LD, coinciding with the beginning of summer, we observed a clear induction of *FaCO* in both cultivars that correlates with higher *FaFT1* expression in leaves (Figure 1B). In ‘Chandler,’ *FaCO* upregulation in leaves parallels a drop in *FaTEM* expression in the same tissue (Figure 1B). Although not statistically significant, a similar trend in *FaTEM* expression was observed in ‘Selva.’ As proposed in *F. vesca* (Koskela et al., 2012; Rantanen et al., 2014), in cultivated strawberry FaFT1 induced in leaves might act as a long-distance signal and move to the apical meristems. However, in the presence of functional FaTFL1, the overall effect is floral repression and promotion of the vegetative state, as reflected by the low transcript level of the floral markers *FaLFYa* and *FaAP1* (Figure 1A). Noteworthy, the most prominent change in gene expression under LD in the two cultivars was the strong *FaSOC1* induction detected both in leaves and crowns (Figure 1B).

In the transition to winter, under SD, *FaFT2* and *FaFT3* expression showed a sharp peak in ‘Chandler’ leaves. In ‘Selva,’ a moderate similar trend was detected, suggesting the rise in expression of these genes might be transient and could have occurred earlier in ‘Selva’ (Figure 1B). Additionally, under flowering inductive SD, all three *FaFT* genes were induced in crowns of both cultivars, although *FaFT2* induction was only clearly detected in ‘Chandler’ (Figure 1B). *FaFTs* upregulation in the meristems is compatible with them acting upstream of the floral identity genes *FaLFYa* and *FaAP1* and thus promoting floral induction and inflorescence development under SD in cultivated strawberry (Figure 1A).

### *FaCO and FaSOC1* spatial and diurnal expression in cultivated strawberry

In the two *F.* × *ananassa* cultivars analyzed in this study, *FaCO* and *FaSOC1* expression is photoperiodically regulated. LD induced the expression of both genes, suggesting *FaCO* and *FaSOC1* might be involved in the regulation of the seasonal alternation between flowering and runnering in response to day-length. Therefore, we decided to focus our work on these two transcription factors.

**FIGURE 2:**
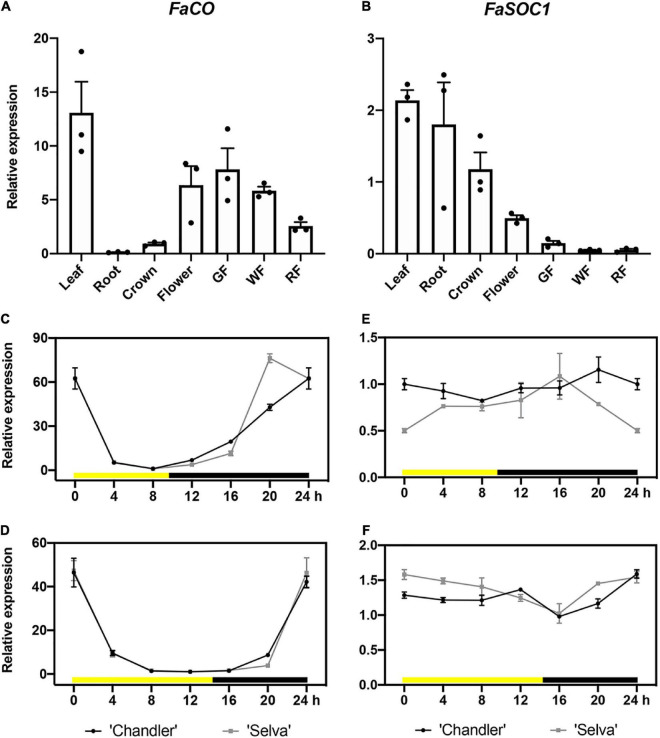
Spatial and diurnal expression of *FaCO* and *FaSOC1* by RT-qPCR. Relative expression levels of *FaCO* **(A)** and *FaSOC1* **(B)** in different tissues or organs. mRNA diurnal rhythms of *FaCO* under SD **(C)** and LD **(D)**. mRNA diurnal rhythms of *FaSOC1* under SD **(E)** and LD **(F)**. The first sample was taken at dawn: 0 h Zeitgeber time (ZT). Relative gene expression levels were normalized to the expression of *FaGAPDH* and crown tissue **(A,B)**, 8 h **(C,D)**, or 16 h **(E,F)** were used as reference samples. Data are means ± SEM of three biological replicates.

First, we analyzed the expression of *FaCO* and *FaSOC1* in different tissues collected under SD from cultivar Chandler. As shown in Figure 2A, *FaCO* expression was specific to the aerial tissues of the plant, with leaves showing the highest *FaCO* transcript levels. This high expression is compatible with FaCO forming part of the photoperiod measurement system in leaves, as described in other species. Elevated *FaCO* expression was also detected in flowers and decreased during fruit development and ripening. In the case of *FaSOC1*, transcripts were detected in all tissues analyzed, although expression was highest in leaf and root, followed by crown, and it was markedly lower in reproductive organs (Figure 2B).

Then, the circadian expression of *FaCO* and *FaSOC1* was analyzed in leaves collected from the SF and PF accessions ‘Chandler’ and ‘Selva’ grown under SD and LD photoperiods (Figures 2C–F). *FaCO* daytime expression rhythm was similar to the one described for its ortholog *FvCO* (Rantanen et al., 2014), showing a peak of expression at the end of the dark period then quickly declining at dawn. The same pattern was observed under LD and SD photoperiods and in the two genotypes analyzed, and thus, the different flowering habits cannot be explained by differences in *FaCO* diurnal pattern of expression.

On the contrary, diurnal *FaSOC1* expression did not show any clear rhythm in none of the photoperiods tested nor cultivar-dependent differences. These results stand in contrast to previous observations by Koskela et al. (2016) in the SD cultivar Honeoye. They did not observe any diurnal oscillation under SD either, but in LD *FaSOC1* was slightly up-regulated in the morning. As occurred in the case of *FaCO*, the differences in flowering behavior between genotypes can’t be explained by different circadian expression patterns of *FaSOC1*.

### Effect of constitutive overexpression of *FaCO* on flowering and runnering in *F.* × *ananassa*

To further investigate the molecular mechanisms involved in the tradeoff between runnering and flowering and the role of *FaCO* in these processes in cultivated strawberry, we generated transgenic plants of the SF cultivar Camarosa ectopically expressing *FaCO* under the control of the CaMV 35S promoter. *FaCO* cDNA was amplified from leaf samples and sequence analysis revealed it corresponds to homoeolog *FxaC_23g53510* from ‘Camarosa’ chromosome Fvb6-2 (Edger et al., 2019). Several putative transgenic shoots from independent transformation events were obtained, from which nine PCR positive lines were acclimated and transferred to the greenhouse for initial evaluation and selection. *FaCO* overexpression was confirmed by RT-qPCR, with transgenic lines showing expression levels 7–80 times higher than the control (Supplementary Figure 2A). Flowering initiation was evaluated in these plantlets coming directly from tissue culture and therefore only one clone per transgenic line was available. A wide variation in the number of days before flowering was found among the nine transgenic lines (Supplementary Figure 2B). With the exception of line CO OverExpressor (COE)-14, all transgenic lines were characterized by less vigor, showing smaller plant size and slightly smaller leaves. Runner capacity was also affected at this stage. Three of the nine transgenic lines evaluated (lines COE-22, -40, and -42) were not able to produce runners, three had an intermediate phenotype (COE-12, -15, and -21) and only three (COE-11, -13, and -14) generated a similar number of runners than the controls, although the severity of this phenotype did not correlate with transgene expression level (Supplementary Figure 2A).

**FIGURE 3:**
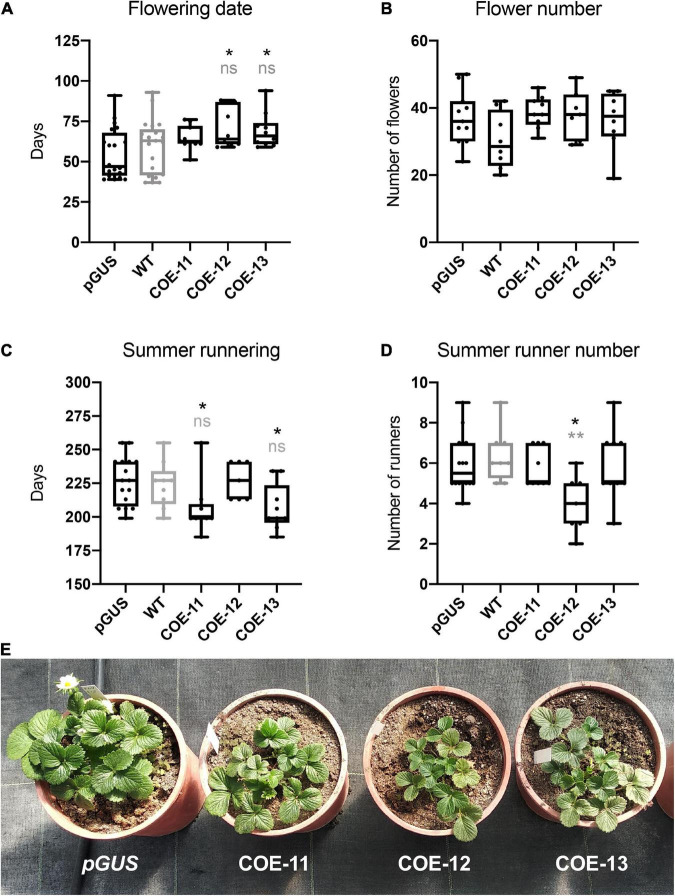
Phenotype of 35S:*FaCO* lines (COE) in comparison to control plants. **(A)** Number of days to flowering from November 20th. **(B)** Total flower number (from November 20th until September 1st). **(C)** Number of days until the first summer stolon. **(D)** Number of runners per plant. **(E)** Phenotype of 35S:*FaCO* lines in comparison to control GUS plants. One representative replicates is shown. Boxes span the 25th and 75th percentiles and the middle line represents the median. Whiskers (T-bars) are the minimum and maximum values. Asterisks indicate significant differences between pGUS (in black) or ‘Camarosa’ wild type (WT; in gray) and 35S:*FaCO* overexpressor plants; statistical significance was determined by ANOVA and Tukey’s test (*P < 0.05, ^**^P < 0.01).

Lines unable to runner cannot be propagated and therefore are lost, hampering further evaluation of the effect of *FaCO* overexpression on runnering capacity beyond the first generation. Among the lines able to runner, COE-11, -12, and -13 were chosen as representative of the phenotypic variability observed in T0 lines and vegetatively propagated for further analyses. A total of nine plantlets per transgenic or control lines (WT and 35S:*GUS*) were grown in a greenhouse under natural light and temperature conditions. The three selected lines maintained a slightly smaller plant size throughout next generations (Figure 3). Winter flower initiation was slightly delayed in COE lines compared to control plants (Figure 3A), although statistically significant differences were only observed between pGUS control and lines COE-12 and 13, which flowered an average of 16 or 14 days later, respectively. Despite this subtle delay in the emergence of the first flowers, no differences in the cumulative number of flowers were observed (Figure 3B). Following the flowering season, runner initiation occurred slightly sooner in lines COE-11 and 13 (Figure 3C), but both of them ended up producing a similar number of runners. On the contrary, line COE-12, that initiated runners at the same time than controls, produced significantly less number (Figure 3D), as already observed during the first season (Supplementary Figure 2A). Altogether, these results point to a marginal and contrasting role of FaCO in the control of meristem fate, at least under the growing conditions used in this work.

### Overexpression of *FaSOC1* delays flowering and promotes vegetative development in *F.* × *ananassa*

*FvSOC1*, the *FaSOC1* ortholog from the diploid relative *F. vesca*, is known to alter the balance between runnering and flowering toward runnering, acting as a central hub where the fate of the meristem is established (Mouhu et al., 2013). To determine whether *FaSOC1* has a similar role in the cultivated strawberry, it was overexpressed in *F.* × *ananassa* cv. Camarosa. *FaSOC1* cDNA used for transformation was amplified from crowns and corresponded to the homoeolog *FxaC_25g18220* from ‘Camarosa’ chromosome Fvb7-2 (Edger et al., 2019). Eight PCR positive lines were selected and transferred to soil, all of them showing markedly higher *FaSOC1* expression in leaves than the control (Supplementary Figure 3A).

In a first-year evaluation, all 35S:*FaSOC1* lines exhibited increased vegetative development except line SOC1 OverExpressor (SOE)-1, the one with lower *FaSOC1* expression (Supplementary Figure 3C). A correlation between transgene expression level and phenotypic severity was observed and thus, lines SOE-20 and -21, the higher overexpressors, also showed the most severe phenotype. As occurred in 35S:*FaCO* T_0_ plants, a wide variability in flowering time was found among the eight 35S:*FaSOC1* lines. Four of them were late flowering, with SOE-7, -9, and -20 being particularly late, as they flowered at least 60 days later than the controls (Supplementary Figure 3B).

**FIGURE 4:**
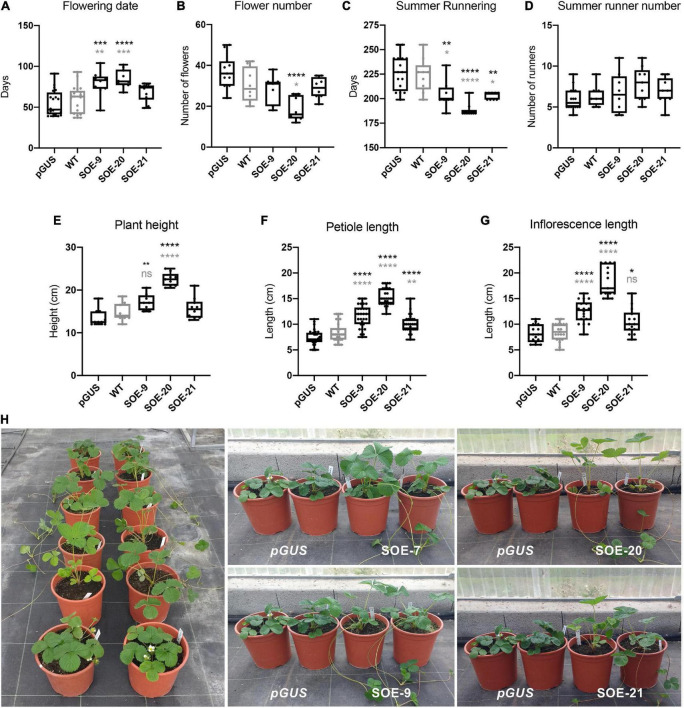
Phenotype of 35S:*FaSOC1* lines (SOE) in comparison to control plants. **(A)** Number of days to flowering from November 20th. **(B)** Total flower number (from November 20th until September 1st). **(C)** Number of days until the first summer stolon. **(D)** Number of stolons until September 20th. **(E)** Plant height. **(F)** Length of leaf petioles. **(G)** Length of inflorescence peduncle. Boxes span the 25th and 75th percentiles and the middle line represents the median. Whiskers (T-bars) are the minimum and maximum values. Asterisks represent significant differences between pGUS (in black) or ‘Camarosa’ wild type (WT; in gray) and 35S:*FaSOC1* over-expresser plants; Statistical significance was determined by ANOVA and Tukey’s test (*P < 0.05, **P < 0.01, ***P < 0.001, ****P < 0.0001). **(H)** Phenotype of 35S:*FaSOC1* lines in comparison to control GUS plants. Two representative replicates are shown. Pictures were taken in November.

Lines SOE-7, -9, -20, and -21 were clonally propagated from runner cuttings. Nine plants per line, except SOE-7, were subjected to further molecular and phenotypic evaluation during the growing season, from December to September, in successive years. The number of days until the emergence of the first flower and the total number of flowers per plant were scored, showing *FaSOC1* overexpression had an evident impact on flowering induction (Figure 4). All 35S:*FaSOC1* lines flowered later than the controls (Figure 4A). Lines SOE-9, -20, and -21 flowered an average of 20, 25 and 9 days later than the WT, which in turn flowered 4 days later than the pGUS control, although statistically significant differences were only observed between both controls and SOE-9 and -20. Additionally, SOE-20 presented a significantly reduced number of flowers compared to control lines.

A very distinctive phenotype of the four SOE-7, -9, -20, and -21 lines was the production of runners in winter, immediately after separation from mother plants and before floral induction (Figure 4H). During summer LD, the three evaluated lines also produced runners significantly earlier than the controls (Figure 4C). Control lines started runners 223 or 227 days after planting while transgenic lines produced runners after 188–203 days. Although transgenic lines generated a slightly higher number of runners per plant, no significant differences were observed. The more vigorous appearance of 35S:*FaSOC1* lines was not a consequence of higher amount or larger leaves but rather more erect and taller plants. Although all of them were higher than the controls (Figure 4H), statistically significant differences were only observed in SOE-9 and -20 (Figure 4E). This phenotype was the reflection of considerably longer leaf petioles displayed by all SOE lines (Figure 4F), which also presented longer inflorescence peduncles (Figure 4G).

Ectopic and constitutive overexpression of *FaSOC1* negatively affected floral and fruit development (Supplementary Figure 4), increasing the incidence of malformed fruits and aborted flowers. Flowers within the inflorescences were more compact and presented defects in the sepal and petal whorls. Sepals were bigger whereas petals, although present, were reduced in size and senesced prematurely compared to the WT. Fruits were in general smaller and in the majority of cases did not develop into fleshy fruits. Line SOE-20, the one with higher transgene overexpression and stronger vegetative phenotype, was also the most affected in fruit development. The majority of SOE-20 fruits got completely dry in the green stage and only a small percentage developed further into dry pinkish fruits that remained attached to the plant. Line SOE-9 presented an intermediate phenotype and developed few wild type ripe fruits, while about half the fruits in line SOE-21 ripened into red fleshy fruits. Noticeably, achenes remained green in all three transgenic lines (Supplementary Figure 4).

Taken together, the photoperiodic pattern of *FaSOC1* expression and the phenotype of 35S:*FaSOC1* lines, point to *FaSOC1* acting as a negative regulator of flower induction and promoter of vegetative growth in cultivated strawberry.

### Flowering-related genes were differentially expressed in 35S:*FaSOC1* lines

**FIGURE 5:**
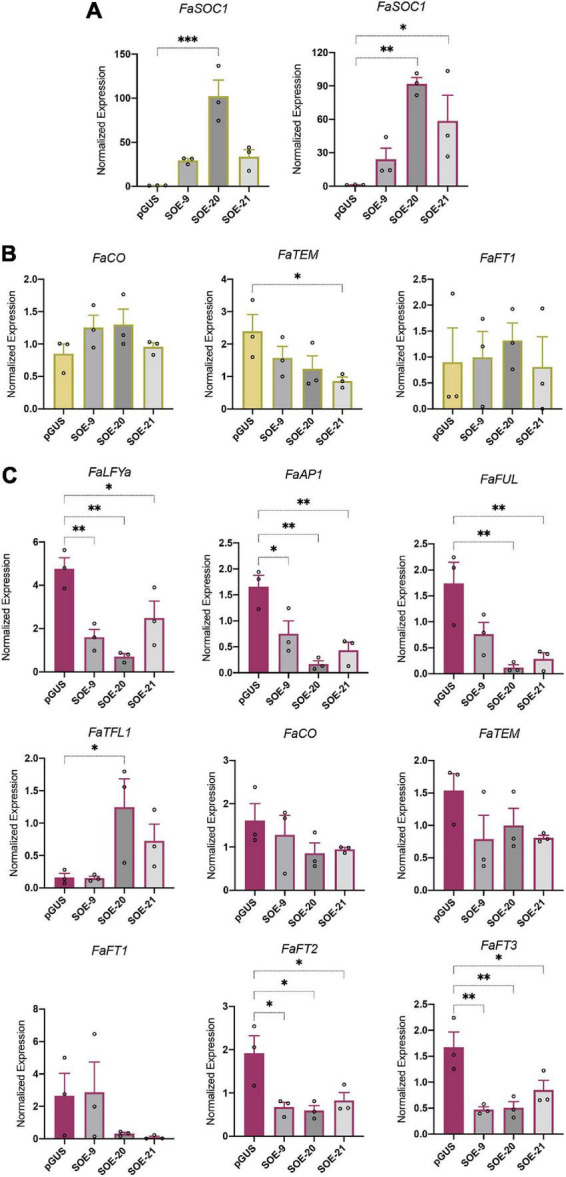
Gene expression analysis in 35S:*FaSOC1* lines (SOE). **(A)** *FaSOC1* overexpression in leaves (green, left panel) and crown (purple, right panel) of three independent SOE lines. **(B)** *FaCO, FaTEM and FaFT1* expression in leaves of SOE lines. **(C)** Flowering time gene expression in crowns from SOE lines. All samples were collected under natural SD (9 h52 min/14 h17 min day/night; average maximum and minimum temperature from previous 4 weeks: 21.1/10.6°C) conditions at ZT3. Graphs show the average of normalized values from three biological replicates ± SEM. In each bar, dots represent the normalized value of each biological replicate. *p < 0.05, **p < 0.01, ***p < 0.001.

Since the transgenic plants overexpressing *FaSOC1* have a late flowering phenotype, the expression of flowering time genes, including *FaCO*, *FaTEM*, *FaFT1-3*, *FaTFL1*, *FaLFYa*, *FaAP1*, and *FaFUL*, was tested in all of them. Expression analysis were performed in young leaf and crown tissue collected under natural SD conditions. Overexpression of *FaSOC1* in leaf and crown of transgenic plants used in these experiments was confirmed by RT-qPCR (Figure 5A). As observed in the first season (Supplementary Figure 3A), SOE-20 was the line with highest transgene expression, followed by SOE-21 and then SOE-9.

*FaCO* expression levels were similar in 35S:*FaSOC1* and control plants in the two tissues analyzed (Figures 5B,C), supporting the model proposed in *F. vesca* that places *FvCO* upstream of *FvSOC1* (Kurokura et al., 2017). However, *FaTEM* was downregulated in leaves of all three SOE lines, although differences were only statistically significant in SOE-21. *FaTEM* expression in crowns, whereas not as evident as in leaves, also tended to be downregulated in the three transgenic lines (Figures 5B,C). The only *FaFT* family member detected in leaves of these SD grown plants was *FaFT1*, but its expression level was low and not affected by *FaSOC1* overexpression (Figure 5B).

The expression of the floral meristem genes *FaLFYa*, *FaAP1*, and *FaFUL* was significantly downregulated in *35S:FaSOC1* crowns compared to the control (Figure 5C), in agreement with their respective phenological stage. Whereas controls were flowering, SOE lines showed active vegetative development at the time of sampling. Additionally, *FaTFL1* expression was induced in lines SOE-20 and -21 (Figure 5C), supporting *FaTFL1* activation downstream of *FaSOC1* also happens in *F.* × *ananassa* as previously described in *F. vesca* (Mouhu et al., 2013).

Along with expression changes in floral meristem identity genes, the most dramatic changes in gene expression observed in 35S:*FaSOC1* crowns affected *FaFT2* and *FaFT3*, with transcript levels of both genes greatly reduced in all SOE lines (Figure 5C). In contrast, *FaFT1* expression was similar in SOE and control lines (Figures 5B,C).

### *FaSOC1* overexpression does not activate gibberellin biosynthesis to promote vegetative growth

‘Camarosa’ plants overexpressing *FaSOC1* showed a characteristic elongated phenotype and initiated runners during winter short days, as previously observed in *F. vesca* overexpressing *FvSOC1* (Mouhu et al., 2013), resembling the effect of GA-treated plants (Thompson and Guttridge, 1959; Hytönen et al., 2008). Indeed, in *F. vesca*, FvSOC1 positively regulates the expression of the GA biosynthetic genes *FvGA20ox* and *FvGA3ox* required for runner differentiation (Mouhu et al., 2013). In order to evaluate if *FaSOC1* modulates GA metabolism, the expression of *FaGA20ox2, FaGA20ox4*, *FaGA3ox1, FaGA3ox2*, and *FaGA2ox1* was analyzed in leaves and crowns of SOE lines grown under SD.

**FIGURE 6:**
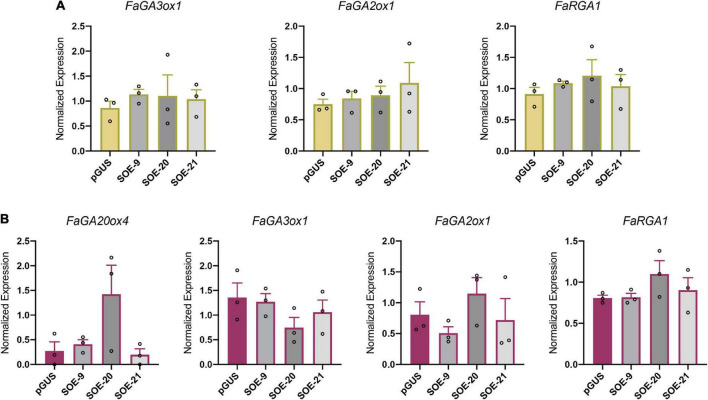
Overexpression of *FaSOC1* does not alter the expression of GA metabolism and signaling related genes. **(A)** *FaGA3ox1*, *FaGA2ox1*, and *FaRGA1* expression in leaves of three independent SOE lines. **(B)** *FaGA20ox4*, *FaGA3ox1, FaGA2ox1*, and *FaRGA1* expression in crowns from SOE lines. All samples were collected under SD. Graphs show the average of normalized values from three biological replicates ± SEM. In each bar, dots represent the normalized value of each biological replicate.

*FaGA20ox2* and *FaGA3ox2* were not detected in any of the tissues analyzed, neither in control nor in transgenic lines. *FaGA20ox4* was not detected in leaves but, although close to detection limits, it could be amplified from crowns. In lines SOE-9 and SOE-21 *FaGA20ox4* transcript level was similar to the control (Figures 6A,B). Only in the most severe SOE-20, *FaGA20ox4* tended to be upregulated but transcript levels were highly variable among the three biological replicates and thus not statistically significant (Figure 6B). *FaGA3ox1* expression in leaves and crowns was similar in SOE and control lines (Figures 6A,B).

As active GA levels are the result of their biosynthesis as well as their deactivation rates, *FaGA2ox1* expression, an oxidase involved in GA deactivation, was also analyzed. *FaGA2ox1* transcripts were detected in leaves and crowns but the expression level, as happened with *FaGA20ox4 and FaGA3ox1*, was not affected by *FaSOC1* overexpression (Figures 6A,B). Hence, our results do not support a role for FaSOC1 as an inducer of active GA accumulation in *F.* × *ananassa*, as was shown for its ortholog FvSOC1 in *F. vesca* (Mouhu et al., 2013).

Alternatively, as a similar constitutive GA response phenotype was described in the suppressor of runnerless (srl) *F. vesca* mutant (Caruana et al., 2018), the vegetative phenotype of SOE lines could also be explained by lower expression of the GA signaling repressor FaRGA1 instead of activation of GA biosynthesis. However, no significant differences in *FaRGA1* expression were detected between the control and transgenic lines (Figures 6A,B), and therefore, the vegetative phenotype of *S*OE lines cannot be explained by neither increased endogenous active GAs levels nor reduced *FaRGA1* expression.

### Transcriptional response of flowering time genes to exogenous gibberellin treatment

**FIGURE 7:**
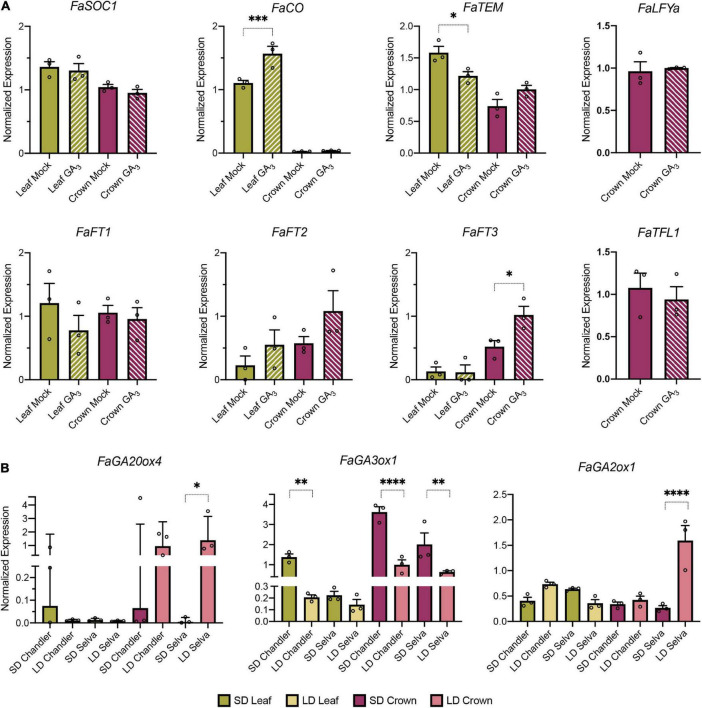
Role of GA in the shift between floral and vegetative development in *F.* × *ananassa.* **(A)** Flowering time gene expression in response to exogenous GA_3_ treatment in leaves (green) and crowns (purple) from SF ‘Camarosa’ plants. **(B)** GA metabolism gene expression in leaves and crowns from the SF cultivar Chandler and the PF cultivar Selva collected under SD and LD. Graphs show the average of normalized values from three biological replicates ± SEM. In each bar, dots represent the normalized value of each biological replicate. *p < 0.05, **p < 0.01, ***p < 0.001, ****p < 0.0001.

Our expression studies indicate that the elongated phenotype and increased runner production observed when *FaSOC1* was overexpressed was not due to a rise on GA biosynthesis as previously reported in *F. vesca* (Mouhu et al., 2013). Alternatively, we hypothesized that *FaSOC1* could be a target gene of GA signaling in *F.* × *ananassa* as it has been shown in *Arabidopsis* (Moon et al., 2003). To test this possibility, *F.* × *ananassa* plants were sprayed with 50 ppm GA_3_ and gene expression analysis was conducted on GA_3_- and mock-treated plants. Since there is strong evidence for negative feedback control of the expression of *GA20ox* and *GA3ox* genes by GA (Hedden and Phillips, 2000), the expression of *FaGA20ox4* and *FaGA3ox1* was analyzed. In our conditions, *FaGA20ox4* expression did not respond to GA application but *FaGA3ox1* was notably down-regulated in GA_3_-treated samples, both in leaves and crowns, indicating that *FaGA3ox1* is involved in the negative feedback regulation of the GA biosynthetic pathway in *F.* × *ananassa* (Supplementary Figure 5). Despite plants were responding to GA treatment, identical *FaSOC1* transcript levels were found in control and treated plants (Figure 7A).

To further investigate the molecular events downstream of GA perception, the expression of other flowering time genes was examined. Transcript accumulation of *FaLFYa*, *FaTFL1*, *FaFT1*, and *FaFT2* did not significantly differ between treated and untreated samples (Figure 7A). In contrast, we observed significant differences in the accumulation of *FaCO*, *FaTEM*, and *FaFT3* (Figure 7A). In leaves, *FaCO* and *FaTEM* expression followed opposite regulation, with GA_3_ mimicking the effect of LD conditions on their expression levels (Figures 1B,7A). *FaCO* levels raised after GA_3_ treatment whereas *FaTEM* dropped in the same samples. In crowns, a two-fold induction of *FaFT3* was observed in response to GA_3_, suggesting that *FaFT3* upregulation in the AXMs might be required for the transition to the vegetative phase in *F.* × *ananassa*.

### Gibberellin metabolism genes are differentially regulated in seasonal flowering and perpetual flowering cultivars in response to photoperiod

In strawberry, active GA levels, together with photoperiod and temperature, regulate the transition to the vegetative phase. In *F. vesca*, the signaling cascade mediating these effects has been partially elucidated, with LD upregulating *FvSOC1* which, in turn, induces *FvGA3ox* and *FvGA20ox* gene expression, resulting in higher GA accumulation (Mouhu et al., 2013). In *F. × ananassa*, GA biosynthesis is required to induce runner production under LD (Hytönen et al., 2009) but, according to our results, its activation is not mediated solely by FaSOC1. Still, GA metabolism genes might be photoperiodically regulated through a FaSOC1 independent pathway. In order to explore how photoperiod regulates GA metabolism in *F.* × *ananassa* and putative differences between cultivars with contrasting flowering habits, *FaGA3ox1, FaGA20ox4*, and *FaGA2ox1* expression was analyzed in ‘Selva’ and ‘Chandler’ under SD and LD natural conditions.

*FaGA3ox1* expression in leaf and crown was promoted by SD (Figure 7B). By contrast, *FaGA20ox4*, which in *F. vesca* is essential for runner production (Tenreira et al., 2017), showed opposite regulation. In leaves, *FaGA20ox4* transcripts were barely detected under any of the photoperiods tested. In SD crowns, *FaGA20ox4* was only detected in one biological replicate from the SF cultivar Chandler (Figure 7B). However, LD conditions considerably upregulated *FaGA20ox4* accumulation in crowns from the two cultivars. Interestingly, in parallel to *FaGA20ox4* induction in LD crowns, a sharp increase in *FaGA2ox1* expression was detected exclusively in the PF cultivar Selva (Figure 7B). This *FaGA2ox1* upregulation merely in the PF cultivar suggests a putative mechanism to regulate continuous flowering in *F*. × *ananassa*, as *FaGA20ox4* induction in LD-grown meristems would be likely counteracted by the high level of *FaGA2ox1* expression, and therefore GA deactivation would neutralize its biosynthesis.

## Discussion

Although the molecular network regulating flowering transition in annual plants, *Arabidopsis* in particular, is well-understood, there has been little research on the molecular mechanisms leading to flower induction in perennial species. Within the past decade, a significant advance in the understanding of the regulation of flowering and runnering in the perennial Rosaceae *F. vesca* has been made, showing substantial differences regarding the role of particular transcription factors compared to what we know from *Arabidopsis* and other annual plants. One of such singularities is the function of the MADS-box transcription factor SOC1. Whereas in annual plants, SOC1 functions as an integrator promoting floral transition and the development of floral organs (Lee and Lee, 2010), strawberry FvSOC1 represses flowering and promotes vegetative growth (Mouhu et al., 2013). The environmental control of flowering in woodland and cultivated strawberry is comparable and therefore, due to the complex genetics of cultivated strawberry, most of the basic knowledge on the molecular events leading to flower induction comes from studies in *F. vesca*, whereas in the octoploid species it is still scarce. The aim of this study was to further advance in the understanding of the genetic control of flowering and runnering in *F.* × *ananassa* and elucidate how well *F. vesca* knowledge is translated to the cultivated strawberry.

### *FaSOC1* has a conserved role as a repressor of flowering in *F.* × *ananassa*

*FaSOC1* expression is detected in a range of vegetative and reproductive tissues, although expression levels are notably lower in flowers and fruits indicative of a major role during vegetative development. No significant diurnal rhythm was observed in its expression neither in SD nor in LD. In contrast, *FaSOC1* expression showed a marked response to day length in both leaf and crown and in ‘Chandler’ and ‘Selva,’ with an average six-fold induction under long photoperiods, again supporting its role during the vegetative phase as described for *F. vesca FvSOC1* (Mouhu et al., 2013). Overexpression of *FaSOC1* in the SD cultivar Camarosa delayed flowering, confirming the role of FaSOC1 as a strong repressor of flowering. At the transcriptional level, *FaSOC1* overexpression downregulates the expression of *FaFT2* and *FaFT3* in crowns, with a concomitant decline in the accumulation of floral identity genes such as *FaAP1*, *FaLFYa*, and *FaFUL* (Figure 5B). Mouhu et al. (2013) showed FvSOC1 activates the transcription of *FvTFL1* and that FvTFL1 function is required for the FvSOC1-induced flower repression in *F. vesca*. In our SOE lines, *FaTFL1* upregulation was not consistent in all transgenic lines (Figure 5B), being only detected in SOE-20 and -21, the ones with higher transgene expression level. A yet uncharacterized SOC1 independent mechanism of *TFL1* regulation has been proposed in *F. vesca* acting at low (<11°C) and high temperatures (>23°C) (Rantanen et al., 2015) or in *F. nilgerrensis* at 11–18°C (Fan et al., 2022), suggesting this other temperature-responsive pathway could be acting in ‘Camarosa’ in our growing conditions. Since the late flowering phenotype was similar in the three transgenic lines (Figure 4A) and it did not correlate with *FaTFL1* expression level, we concluded that in ‘Camarosa,’ *FaSOC1*-dependent *FaFT2* and *FaFT3* repression in crowns might have a major role mediating flowering repression. Additionally, *FaTFL1* activation is only achieved at higher *FaSOC1* transgene levels and do not correlate with the strength of the flowering time phenotype. These results highlight once more the importance of the TFL1/FT balance in the control of meristem fate in strawberry (Gaston et al., 2021) and suggest FaSOC1 modifies this balance to promote the vegetative state in *F.* × *ananassa* mainly through *FaFT2* and *FaFT3* downregulation.

### *FaSOC1* promotion of vegetative development occurs independently of gibberellin biosynthesis

In addition to delaying flowering, overexpression of *FaSOC1* has various effects on growth and development during the vegetative phase. In SF octoploid cultivars, under natural growing conditions, a sharp upregulation of *FaSOC1* expression in leaves and crowns correlates with the induction of runner formation and petiole elongation, which are normal photoperiodic responses to increasing day lengths (Guttridge and Thompson, 1964). We show here that *FaSOC1* is involved in the regulation of both processes as petioles are significantly longer and the emergence of runners takes place 3–6 weeks earlier in 35S:*FaSOC1* than in control lines, although the final count of runners is only slightly increased at the end of the season (Figure 4). Similar morphological and physiological effects are observed after exogenous GA treatment (Thompson and Guttridge, 1959; Guttridge and Thompson, 1964; Hytönen et al., 2009). In fact, the phenotype of *F. vesca* plants overexpressing *FvSOC1*, comparable to that in our 35S:*FaSOC1* lines, is due to an upregulation of *GA3-ox* and *GA20-ox* genes, leading to increased GA accumulation (Mouhu et al., 2013). However, we could not confirm a similar mechanism in ‘Camarosa’ SOE lines, as none of the tested genes in the GA metabolic pathway presented differential expression compared to the control (Figure 6). The activity of the GA biosynthetic enzyme GA20ox is of particular importance in determining GA concentration in many plant species. In *F. vesca, FvGA20ox4* activation in AXM under LD has been shown to be required for stolon development. Whereas at 18°C this activation occurs *via* an FvSOC1-dependent photoperiodic pathway (Mouhu et al., 2013; Tenreira et al., 2017; Andrés et al., 2021), *FvGA20ox4* upregulation also occurs independently of FvSOC1 (Andrés et al., 2021; Fan et al., 2022). The latter situation is therefore in line with our observations in cultivated strawberry. Although *FaSOC1* overexpression did not affect that of *FaGA20ox4*, this gene showed a marked photoperiodic response in crowns in response to LD (Figure 7B), suggesting it plays a critical role in the accumulation of active GA required for inducing the vegetative phase, in agreement with its role in the diploid species. Furthermore, an interesting difference between the PF cultivar Selva and the SF cultivar Chandler was observed in *FaGA2ox1* expression, coding for a GA inactivation enzyme (Figure 7B). *FaGA2ox1* transcript accumulation in ‘Chandler’ was low under SD and LD photoperiods, and equivalent to that in ‘Selva’ under SD. However, a marked upregulation of *FaGA2ox1* was detected in ‘Selva’ under LD. Increased FaGA2ox1 activity is expected to decrease the levels of bioactive GAs, negatively affecting runner production and promoting branch crown development, aiding continuous flowering in ‘Selva.’

In an attempt to explain the constitutive GA response in SOE lines, we considered the possibility of *FaSOC1* being a mediator of GA signaling, activated downstream of GA perception, similar to Arabidopsis *SOC1*, which is induced by GAs (Lee and Lee, 2010). We could not confirm this hypothesis as, in our studies, *FaSOC1* expression was equivalent in GA-treated and mock plants (Figure 7A). However, although plants were sensing and responding to GA, a weakened response to exogenous GA cannot be completely ruled out under our photoperiodic conditions, as daylight was slightly below 12h at the time of the first application. Previous studies showed *F.* × *ananassa* plants are able to respond to exogenous GA treatment under SD (Guttridge and Thompson, 1959), but also a reduced sensibility has been observed in short compared to long photoperiods (Hytönen et al., 2009).

Considering *FaSOC1* expression does not respond to GA treatment, we therefore hypothesized that GA signaling and FaSOC1 might have common target genes that would account for the vegetative phenotype observed in SOE lines. Among the flowering genes evaluated in this work, *FaTEM1* expression is downregulated in leaves of GA treated and 35S:*FaSOC1* plants. Notably, a similar trend was observed in ‘Selva’ and ‘Chandler’ grown under LD (Figure 1B). The fact that *FaTEM* was downregulated in different scenarios, all of them promoting vegetative development, pointed to a putative role of this factor in the repression of flowering in *F.* × *ananassa.* TEM proteins have been proposed to act as regulators of the juvenile vegetative phase in species as diverse as antirrhinum, *Arabidopsis* or olive (Sgamma et al., 2014), including another perennial Rosaceae such as loquat (Peng et al., 2021). In *Arabidopsis, TEM1* represses photoperiodic flowering at early stages of vegetative growth, then its expression levels progressively decay throughout development to reach a minimum at the time of floral transition, thus allowing *FT* activation by CO (Castillejo and Pelaz, 2008). Recently, it has been shown that heterologous overexpression of apple *MdTEM* in *F. vesca* H4 leads to *FvFT1* induction, whereas the opposite effect is observed in RNAi-*MdTEM1* lines (Dejahang et al., 2022). In *35S:MdTEM1* and *35S:MdTEM2* lines, generated in the *fvtfl1* mutant background of H4, elevated *FvFT1* expression levels in leaves correlated with an extremely early flowering (Dejahang et al., 2022), phenocopying *FvFT1* overexpression in H4 (Rantanen et al., 2014). However, in a *TFL1* WT genotype, *FT1* upregulation in leaves would be expected to induce TFL1 in the SAM and repress flowering.

### *FaCO* overexpression does not prevent flowering under short days in *F.* × *ananassa*

In *F. vesca* H4, FvCO is required for *FvFT1* induction in leaves in response to long photoperiods, functioning as a strong promoter of flowering under LD as there is no functional FvTFL1 (Kurokura et al., 2017). However, in SF genotypes carrying a functional TFL1, the role of CO in the photoperiodic control of the alternance between vegetative and reproductive development needs further investigation. According to the model proposed in *F. vesca*, and considering ‘Camarosa’ harbors a functional allele of the flowering repressor FaTFL1, an expected outcome of *FaCO* overexpression in ‘Camarosa’ was the activation of the FaFT1-FaSOC1-FaTFL1 module and consequently, flowering repression and promotion of vegetative development. As shown in Figure 1, in *F.* × *ananassa*, we observed a correlation in the upregulation of FaCO and FaFT1 in leaf, and FaSOC1 and FaTFL1 in crowns by LDs. However, overexpression of *FaCO* in SF ‘Camarosa’ only causes a subtle delay on flowering time and a similar discrete advance of runnering (Figure 3A), resulting in a slightly extended vegetative phase. These results might indicate the existence of an additional factor that prevents vegetative development under SD even in the presence of elevated FaCO. A putative candidate could be FaTEM, as it has been shown to repress *FaFT1* expression (Dejahang et al., 2022) and it is upregulated in response to SD (Figure 1A). As a result, elevated FaTEM expression levels would not allow *FaFT1* activation by FaCO. Additionally, in contrast to the moderate effect of FaCO in prolonging the vegetative phase, a negative effect of *FaCO* overexpression on runner capacity was detected in most of the T_0_ transgenic lines and further observed in COE-12 (Figure 3D) in the following season. Although T_0_ phenotypes might be affected by the fact that those plants come directly from *in vitro* culture and maybe not related to transgene expression, the possibility of FaCO negatively affecting runner initiation should not be discarded and, in that case, the phenotypes of the three selected runnering lines might not fully reflect FaCO function. In depth characterization of 35S:*FaCO* transgenic lines awaits in order to elucidate the molecular mechanisms regulating FaCO effects on flowering and runnering.

### Expression analysis of *FaFT* genes suggest functional diversification and specific roles in the regulation of flowering and vegetative development

In this work we propose a role for FaCO and FaSOC1 as weak and strong repressors, respectively, of the photoperiodic flowering pathway in *F.* × *ananassa*. Additionally, our expression analysis performed in the SD and PF cultivars, ‘Chandler’ and ‘Selva’ respectively, shed some light into the molecular mechanisms leading to flower induction in both of them. In many different species, *FT* genes have been shown to be major components of the florigen, a graft-transmissible signal produced in the leaves that induces flowering at the shoot apex in response to inductive photoperiods (Andrés and Coupland, 2012). In addition to regulating flowering, FT genes have been implicated in a range of physiological processes, such as promotion of vegetative development in poplar (Böhlenius et al., 2006), tuberization or bulbing in potato and onion, respectively (Navarro et al., 2011; Lee et al., 2013), or more pleiotropic roles as general regulators of growth, like in tomato or maize (Lifschitz et al., 2006; Danilevskaya et al., 2011). In the strawberry genome, three *FT* homologs have been identified (Nakano et al., 2015), however there is still scarce knowledge about the physiological process controlled by each FaFT member, or if variations in their expression pattern are able to explain differences in the vegetative and flowering responses of SD or PF cultivars. Most recent studies overexpressing *F. vesca FvFT2* in *F. vesca* and *F.* × *ananassa* suggest that FaFT2 might act as florigen under SD in cultivated strawberry (Gaston et al., 2021).

In SF cultivars like ‘Chandler,’ floral induction usually takes place at late fall, when diurnal light periods and temperatures become lower, whereas in PF cultivars as ‘Selva,’ flowers are initiated continuously throughout the growing season from spring until late autumn. The length of the flowering period is also variable among cultivars within the SF or PF habits and dependent on genetic and environmental conditions (Stewart and Folta, 2010; Heide et al., 2013; Labadie et al., 2019). In the present study, we have conducted an expression analysis of the three *FaFT* homologs in crowns and leaves of *F.* × *ananassa* ‘Selva’ and ‘Chandler’ grown under natural conditions and collected in SD (December 21st) or LD (June 21st). Under floral promoting SD, higher expression levels of the three *FaFT* homologs, with the exception of *FaFT2* in ‘Selva,’ are observed in crowns, and correlate with the induction of floral identity genes such as *FaAP1* and *FaLFY* and downregulation of the floral repressor *FaTFL1* (Figures 1A,B). *FaFT1* and *FaFT3* upregulation in shoot tips under floral inductive conditions have been previously described in other octoploid cultivars (Nakano et al., 2015; Koembuoy et al., 2020). SD specific *FaFT2* induction in shoot tips was also documented by Nakano et al. (2015) after 7 days of photoperiod treatments, although no differences were observed after 21 days. Interestingly, *FaFT2* and *FaFT3* induction in crowns is abolished when *FaSOC1* is overexpressed (Figure 5B), suggesting SD dependent *FaSOC1* downregulation (Figure 1) is required to allow activation of the floral promoter *FaFT* genes. Alternatively, FaSOC1 might directly repress FaFT2 and FaFT3 in meristems.

Gene expression changes in crowns are expected to occur in response to a long-distance signal coming from leaves (Colasanti and Sundaresan, 2000; Andrés and Coupland, 2012). Accordingly, we observed SD photoperiods induce the accumulation of *FaFT2* and *FaFT3* transcripts in leaves, being more evident in SF ‘Chandler’ than in PF ‘Selva’ at the time sampling was performed (Figure 1B). Upregulation of *FvFT3/FaFT3* by SD was not detected in leaves in previous studies by Nakano et al. (2015) or Gaston et al. (2021). In this latter study, *FaFT2* expression was equivalent in SD and LD leaf samples from the *F.* × *ananassa* Japanese cultivar Nyoho. In contrast, Gaston et al. (2021) detected a transient upregulation of *FvFT2* under SD conditions in a PF, but not in a SF, *F. vesca* genotype. In their monthly time-course performed from June to November under natural environmental conditions, *FvFT2* peaked in October. The observed discrepancies might be due to natural variation among genotypes. Alternatively, peaks might be easily missed due to their transient nature. *FaFT2* induction in PF ‘Selva’ could have already happened when we sampled in December, whereas *FvFT2* rise in the SF cultivar used in Gaston et al. (2021) could have taken place after their last sample in November. According to our qPCR data, upregulation of *FaFT2* and *FaFT3* in leaf is compatible with both of them having partially overlapping roles as florigens. Indeed, *FvFT2* has been already shown to function as a strong floral promoter when overexpressed in *F. vesca* and *F.* × *ananassa* (Gaston et al., 2021; Sabbadini et al., 2021). In the same study, *FvFT3* overexpression only caused a modest advance of flowering in *F. vesca*. In contrast, when *FvFT3* is overexpressed in *F.* × *ananassa*, it promotes the vegetative state of AXM and thus runner development (Gaston et al., 2021; Sabbadini et al., 2021). This apparently contradictory result, with *FaFT3* expression in leaves associated with floral inductive conditions, and the promotion of runner production when overexpressed, raised the possibility of FaFT3 fulfilling different roles, as floral inducer in leaves and SAM and as runner promoter in AXM. In *F.* × *ananassa*, GA is a key signal determining bud fate and needed for runner initiation (Hytönen et al., 2009). Therefore, we quantified *FaFT1-FaFT3* expression in GA-treated samples. Interestingly, *FaFT1* and *FaFT2* expression do not respond to GA, but a two-fold *FaFT3* induction is observed specifically in crowns after exogenous GA application (Figure 7A), supporting a key role for FaFT3 in the regulation of the vegetative developmental fate of AXM downstream GA perception.

As for *FaFT1*, its expression pattern follows opposite photoperiodic regulation in crowns compared to leaves, suggesting it might also fulfill tissue specific roles. Similarly to *F. vesca FvFT1* (Koskela et al., 2012), *FaFT1* is induced in leaves under LD, promoting the vegetative state of plant meristems. However, under SD, when *FaTFL1* expression drops in the meristems, *FaFT1* can act as floral inducer in crowns as previously shown in *F. vesca* H4 in LD or when *FvFT1* is overexpressed (Rantanen et al., 2014).

Altogether, our results suggest the three *F.* × *ananassa* FT proteins have overlapping and particular roles in the regulation of the alternance between flowering and vegetative development. FaFT2 is proposed as the florigenic signal transiently produced in leaves under SD, which likely exerts its function redundantly with FaFT3. At the SAM, *FaFT1-3* induction under SD indicates overlapping roles in the promotion of flowering transition. Additionally, the transition to the vegetative phase would require upregulation of FaFT1 and FaFT3 in leaf and AXMs, respectively.

Overall, this work supports that *F.* × *ananassa* FaSOC1 has retained FvSOC1 role as repressor of flowering and promoter of vegetative development. However, signaling events downstream FaSOC1 show particularities compared to *F. vesca* that hinders direct transfer of knowledge acquired in the diploid species to cultivated ones. On the contrary, FvCO has been shown to promote flowering while repressing runner development in *fvtfl1 F. vesca* accessions, whereas our study suggests that *FaCO* responds to LD photoperiods as in *F. vesca* with a different outcome, as flowering was marginally delayed in COE lines. Comparison of FvCO and FaCO protein sequences did not reveal substantial differences able to explain the contrasting phenotypes in the two *Fragaria* species. A total of 13 aminoacid substitutions were found in the coding sequence, 6 non-synonymous, but none of them affected conserved residues (Supplemental Figure 6). The presence of a functional *FaTFL1* allele in the SF cultivar Camarosa and COE lines is an obvious difference with reported studies in F. vesca and if its activation occurs downstream of FaCO, as it has been suggested for FvTFL1, a more dramatic repression of flowering was expected. The subtle negative effect on flowering of *FaCO* overexpression and the contrasting effects on runner initiation suggest the existence of putative additional factors that counterbalance its activity, particularly under SD conditions.

Moreover, our results provide a groundwork for detailed characterization of factors that may play a critical role in the tradeoff between flowering and runnering in *F.* × *ananassa*. One of such factors is FaTEM, which expression pattern is compatible with a role as repressor of vegetative development under SD, and thus promoter of flowering, likely through preventing FaCO-dependent *FaFT1* activation in leaves. Additionally, LD induction of *FaGA2ox1* in crowns specifically in PF ‘Selva’ suggests a putative mechanism to lower active GA levels in PF genotypes in order to enable successive rounds of flowering. Lastly, detailed expression of *FaFT* genes suggest the three homologs from *F.* × *ananassa* might have overlapping roles inducing flowering in the apical meristem under SD, but also functional divergence in the case of FaFT1 and FaFT3 is proposed. Although further studies are needed to clarify these mechanisms, our work broadens our knowledge on the regulatory pathways controlling flowering and vegetative growth in cultivated strawberry in response to photoperiod, and how these compare with flowering regulation in annual plants or the diploid relative *F. vesca*.

## Data availability statement

The data presented in this study are deposited in the GenBank repository, accession numbers ON996971 *(FaCO)* and ON996972 *(FaSOC1)*.

## Author contributions

JM-A, CC, and IA designed the experiments. JS-S provided plant materials and participated in the design of experimental work. JM-A, CP, and CC carried out the experimental work and statistical analyses. IA and CC supervised the work and wrote the manuscript. All authors commented on and accepted the final manuscript version.
