# Supplementary material for: Role of FaSOC1 and FaCO in the seasonal control of reproductive and vegetative development in the perennial crop Fragaria × ananassa
Source: Front Plant Sci. 2022 Aug 17;13:971846. doi: 10.3389/fpls.2022.971846 (PMC9428485; doi:10.3389/fpls.2022.971846)
Supplement: Supplementary file 1 [file Data_Sheet_1.docx]

Supplementary Material

# Supplementary Table 1. List of primers used in this study. Primers used for qPCR were designed to amplify all *F. x ananassa* ‘Camarosa’ homeologs annotated in the *Fragaria x ananassa* ‘Camarosa’ Genome Assembly v1.0 & Annotation v1.0.a1 (Edger et al., 2019). For simplicity, the gene code corresponding to each *F. vesca* homolog (*Fragaria vesca* Whole Genome v4.0.a2 Assembly & Annotation. Li et al, 2019) is provided.

| **Gene** | ***F.vesca* homolog** | **Forward primer name** | **Forward primer sequence** | **Reverse primer name** | **Reverse primer sequence** | **Purpose** | **Reference** |
| --- | --- | --- | --- | --- | --- | --- | --- |
| *FaAP1* | FvH4_4g29600 | ccm46 | AGCTACAGAGGAAGGAGAAGGC | ccm47 | TTTGCTGCTGCTGCTCCCAG | qPCR | This study |
| *FaCO* | FvH4_6g45860 | RT-FaCO1-F | CTCCGGCTGTCAGATAATGG | RT-FaCO1-R | AGGAAGCAGCCTCATCTTCG | qPCR | This study |
|  |  | F_COfull_Sal I | AGTCGACATGTTGAAAGAGGAGAGCAA | R_COfull_Xba I | ATCTAGACTAGTACGAAGGAACAATGC | cDNA cloning | This study |
| *FaFT1* | FvH4_6g00090 | ccm127 | TATGAAAGTCCACGGCCAAC | ccm128 | ACACAGTTTGCCTTCCCAAC | qPCR | Nakano et al. (2015) |
| *FaFT2* | FvH4_4g30710 | ccm129 | ACTCGGTGGCTTGTGTTTTC | ccm130 | ATCACTCTCCCGACGACAAG | qPCR | Nakano et al. (2015) |
| *FaFT3* | FvH4_3g09870 | ccm131 | AGCCGTTCACCAAGTCTGTG | ccm132 | GTGGACAACATGAGAAGGTTTG | qPCR | Nakano et al. (2015) |
| *FaFUL* | FvH4_5g13500 | ccm141 | TGCCCTTCTACCTCAGGCAT | ccm142 | CGGAAGGAGCGTGTTACTGTT | qPCR | This study |
| *FaGA2ox1* | FvH4_3g16760 | ccm137 | TTTGGAGAGCACACAGACCC | ccm138 | TACTGGTCTGGAGGCACTGA | qPCR | This study |
| *FaGA3ox1* | FvH4_6g30780 | ccm135 | CTAGCAGGGAGGCTGATGTG | ccm136 | CCCGACTAGGATCCGGACAA | qPCR | This study |
| *FaGA3ox2* | FvH4_2g30040 | FaGA3ox2-F | AGACACTTCCCTCGTCACCA | ccm82 | AAGCGTTGGATCGAGTTCAC | qPCR | ccm82: Mouhu et al. (2013) |
| *FaGA20ox2* | FvH4_7g12600 | FaGA20ox2-F | GACAACGAAAAGCCATGCCC | FaGA20ox2-R | CTTCGGAGACAACCGAAGCA | qPCR | Mouhu et al. (2013) |
| *FaGA20ox4* | FvH4_2g35050 | ccm125 | CGACAAACCAAACGAGGTCC | ccm126 | GGCCAAATGAAACCACTGGG | qPCR | This study |
| *FaLFYa* | FvH4_5g09660 | FaLFYa-F | AGTCTGCCATCCGAGCTGAG | FaLFYa-R | TCTTGTTGCACCGGCTCCTC | qPCR | This study |
| *FaRGA1* | FvH4_4g34110 | ccm139 | GAGGAGAACGACGGGTGTTT | ccm140 | AGTGAGTCATCACCGCTTGG | qPCR | This study |
| *FaSOC1* | FvH4_7g12700 | RT-FaSOC1-F | CCAAGCCAATAACAAAGTTGC | RT-FaSOC1-R | TCCCAATAGTTTCCGTTTCG | qPCR | This study |
|  |  | F_SOC1full_Sal I | AGTCGACATGGTGAGAGGCAAAACC | R_SOC1full_Xba I | ATCTAGACTAGTGCTTCGATCTCCTTT | cDNA cloning | This study |
| *FaTEM* | FvH4_4g28030 | ccm116 | CCCAACACAACAACAAGCTCTCATC | ccm117 | CCTCGATGCCGTTCTCGGA | qPCR | This study |
| *FaTFL1* | FvH4_6g18480 | ccm83 | CTGGCACCACAGATGCTACA | ccm84 | AACGGCAGCAACAGGAAC | qPCR | Mouhu et al. (2013) |
| *FaDBP* | FvH4_3g25890 | qDBP-F | TTGGCAGCGGGACTTTACC | qDBP-R | CGGTTGTGTGACGCTGTCAT | qPCR | Galli et al. (2015) |
| *FaGAPDH* | FvH4_4g24420 | qGAPDH-F | TCCATCACTGCCACCCAGAAGACTG | qGAPDH-R | AGCAGGCAGAACCTTTCCGACAG | qPCR | Salvatierra et al. (2010) |

Edger, P.P., Poorten, T.J., VanBuren, R., Hardigan, M.A., Colle, M., McKain, M.R., et al. (2019). Origin and evolution of the octoploid strawberry genome. Nature genetics 51(3), 541-547. doi: 10.1038/s41588-019-0356-4

Li, Y., Pi, M., Gao, Q. et al. Updated annotation of the wild strawberry Fragaria vesca V4 genome. Hortic Res 6, 61 (2019). <https://doi.org/10.1038/s41438-019-0142-6>

**Supplementary Figure 1.** Plant materials used in this study. **(A)** Picture of complete plants after cleaning all the soil. **(B)** Roots were first sampled and immediately frozen in liquid nitrogen. **(C)** Picture of aerial part of the plant, stem without leaves (1) and crown tissue with SAM and AXMs after removing external leaf tissues and stipules (2). **(D)** Representative image of the vegetative tissues used in this study, (1) root, (2) leaf and (3) crown. **(E)** Closed flower (1), green (4), white (5) and ripe (7) fruit stages used in this study.

**Supplementary Figure 2.** 35S::*FaCO* transgenic plants (COE lines). **(A)** Relative expression of *FaCO* in leaf of T_0_ transgenic lines in comparison to control plants overexpressing GUS. Table below the graph indicates plant size and runnering capacity. ++, indicates size and runner capacity equal to WT Camarosa plants; +, indicates reduced runnering capacity, -, indicates smaller plant size or transgenic lines not able to produce runners. **(B)** Number of days to flowering from the first day in the green-house after acclimation. **(C)** Phenotype of 35S::*FaCO* lines in comparison to control GUS plants.

#

**Supplementary Figure 3.** Phenotype of 35S::*FaSOC1* transgenic plants (SOE lines). **(A)** Relative expression of *FaSOC1* in leaf of T_0_ transgenic lines in comparison to two control lines overexpressing GUS. **(B)** Number of days to flowering from the first day in the green-house after acclimation. **(C-F)** Phenotype of 35S::*SOC1* lines in comparison to control GUS plants.

**Supplementary Figure 4.** Phenotype of 35S::*FaSOC1* transgenic plants (SOE lines). **(A)** Representative morphology of *35S::SOC1* transgenic flowers. **(B, C)** Common inflorescence architecture of 35S::*SOC1* transgenic lines, with flowers with abnormal petals that do not develop into fleshy fruits. **(D-F)** Typical flowers and developing fruits of line SOE-20. Fleshy red fruits are rarely produced in some lines as SOE-21 **(G)**. Typical fruits in lines SOE-20 and SOE-9 **(H, I)**. Green **(J)** and ripe **(K)** fruits in wildtype ‘Camarosa’ plants with details of green and brown achenes, respectively.

**Supplementary Figure 5.** Transcriptional response of *FaGA20ox4* and *GA3ox1* to exogenous GA_3_ treatment.


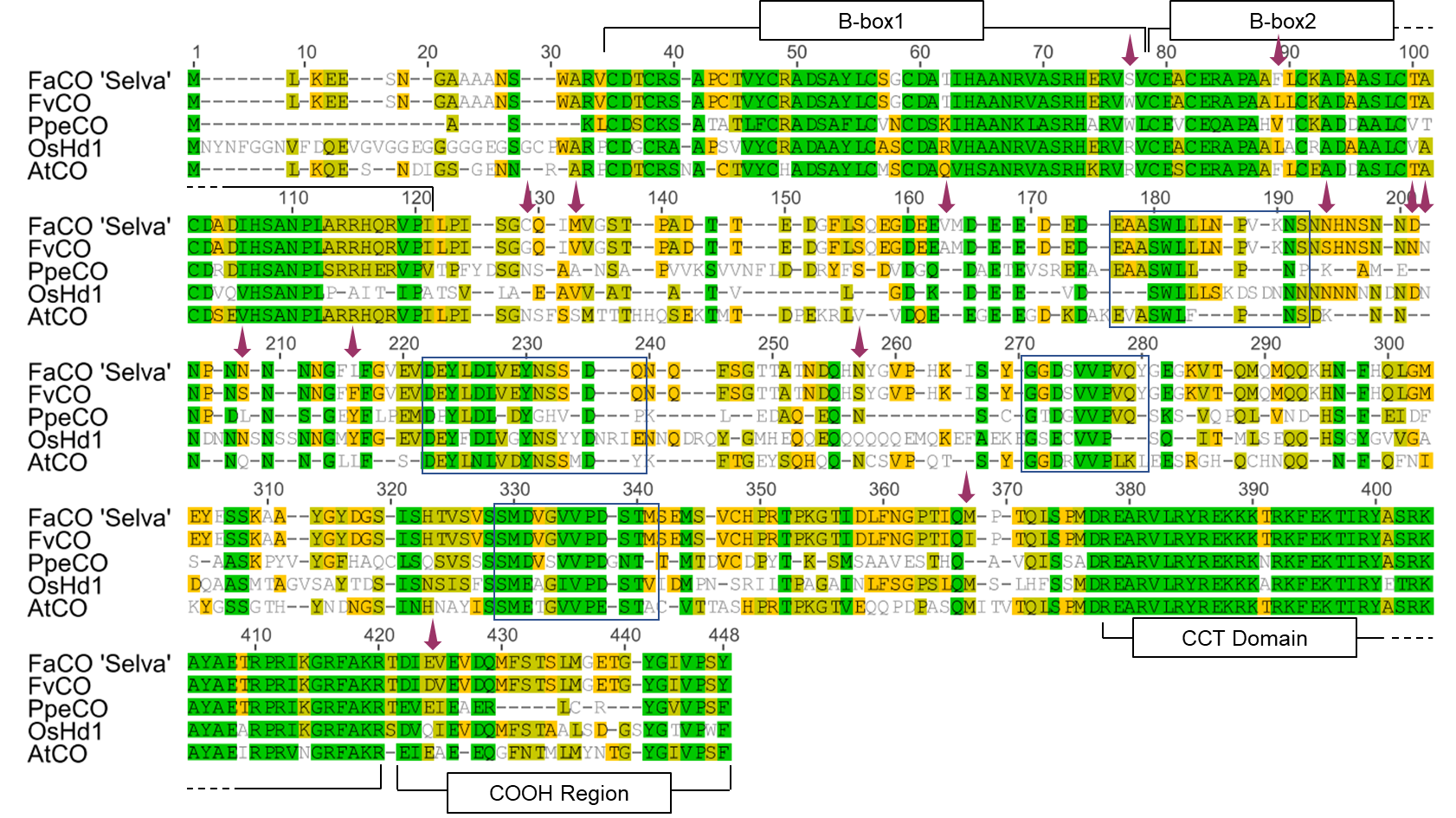


**Supplementary Figure 6.** Alignment of predicted peptide of *Fragaria x ananassa* ‘Selva’ CONSTANS (FaCO ‘Selva’sa) deduced from the cDNA cloned in this work with FvCO (FvH4_6g45860)*, Prunus persica* PpeCO (GenBank: EU939303.1), *Oriza sativa* (GenBank: AB041838.1) and Arabidopsis (AT5G15840). Conserved B-box, CCT and COOH domains are shown. Other small conserved motifs in CO-like proteins are shown in boxes. Purple arrows highlight the 13 divergent residues between *F. vesca* and *F. ananassa* CO proteins. Alignment was performed using Geneious software 7.1.9 (Cost Matrix Blosum62; Gap open penalty: 3; Gap extension penalty: 3).
